# Intellectual disabilities and autism among children with congenital heart defects, Western Australia, 1983–2010

**DOI:** 10.1186/s12887-023-03924-3

**Published:** 2023-03-04

**Authors:** Emine Bircan, Maria D. Politis, Yevgeniya Gokun, Chunqiao Luo, Helen Leonard, Jenny Bourke, Carol Bower, Wendy N. Nembhard

**Affiliations:** 1 Arkansas Center for Birth Defects Research and Prevention and the Department of Epidemiology, Fay W. Boozman College of Public Health, University of Arkansas for Medical Sciences, 4301 West Markham Street, Slot #820, Little Rock, AR 72205-7199 USA; 2 Department of Biostatistics, Fay W. Boozman College of Public Health, University of Arkansas for Medical Sciences, Little Rock, AR USA; 3 Data Science Core, Norris Comprehensive Cancer Center, University of Southern California, Los Angeles, USA; 4 Telethon Kids Institute, The University of Western Australia, Perth, Western Australia Australia

**Keywords:** Congenital heart defects, Intellectual disabilities, Autism

## Abstract

**Background:**

Children with congenital heart defects (CHDs) are at higher risk of developing an intellectual disability. However, severity of intellectual disabilities among this group of children are largely unknown. Our objective was to determine the risk of intellectual disability (ID), ID severity, and autism among children with CHDs.

**Methods:**

We conducted a retrospective cohort study of singleton live births in Western Australia (*n* = 20,592) between 1983 and 2010. Children with CHDs were identified from the Western Australian Register for Developmental Anomalies (*n* = 6563) and infants without CHDs were randomly selected from state birth records (*n* = 14,029). Children diagnosed with ID before 18 years were identified by linkage to statewide Intellectual Disability Exploring Answers database. Odds ratios (OR) and 95% confidence intervals (CI) were calculated from logistic regression models for all CHDs combined and by CHD severity adjusting for potential confounders.

**Results:**

Of 20,592 children, 466 (7.1%) with CHDs and 187 (1.3%) without CHDs had an ID. Compared to children without CHDs, children with any CHD had 5.26 times (95% CI 4.42, 6.26) the odds of having an ID and 4.76 times (95% CI 3.98, 5.70) the odds of having mild/moderate ID. Children with any CHD had 1.76 times the odds of having autism (95% CI 1.07, 2.88), and 3.27 times the odds of having an unknown cause of ID (95% CI 2.65, 4.05) compared to children without CHD. The risk of having autism (aOR 3.23, 95% CI 1.11, 9.38), and unknown cause of ID (aOR 3.45, 95% CI 2.09, 5.70) was greatest for children with mild CHD.

**Conclusions:**

Children with CHDs were more likely to have an ID or autism. Future research should elucidate underlying etiology of ID in children with CHDs.

**Supplementary Information:**

The online version contains supplementary material available at 10.1186/s12887-023-03924-3.

## Background

Every year, worldwide, around 1.4 million infants are born with congenital heart defects (CHDs) [1], the most common type of birth defect with a prevalence of 7.7 per 1000 live births [2]. In Western Australia, the prevalence is similar with about 8–12 cases per 1000 live births annually [3].

As a consequence of significant advancement in pediatric care and surgical techniques [4, 5], the lifespan of children with CHDs has greatly increased in the last several decades [1, 6], with an estimated 85% of children with CHDs living until adulthood [7]. Despite improvements in survival, there is growing evidence that short- and long-term deficits in neurodevelopmental and intellectual outcomes are common in a large proportion of children with CHDs [7–9].

The risk of a poor neurodevelopmental outcome in children with CHD has been shown for specific cardiac defects [10]. However, the specific mechanisms by which children with CHDs have an increased risk of developmental disabilities is not always clear. Possible explanations for increased risk of intellectual disability (ID) among children with CHDs include genetic syndromes, environmental factors [7], brain malformations [11], or brain injury prenatally or after surgery [12], as cardiac surgery in early infancy is often necessary with underlying CHD severity and complexity [7]. Intellectual disabilities can also result from damage occurring prenatally or at birth, during delivery, postnatally or during childhood [10]. CHDs can be part of genetic and other syndromes that also include neurodevelopmental impairments. Conversely, children born with CHDs may have alterations (prenatal as well as postnatal) in the blood flow resulting in compromised delivery of oxygen to the brain, possibly affecting postnatal brain development [7]. Other postulated mechanisms include decreased brain perfusion during early development [13], brain malformations, [14, 15] and post-surgery hypoxemic-ischemic brain injury [12].

Scientific knowledge on neurodevelopment in children with CHDs is still incomplete. Previous studies investigating this association were limited to small sample sizes [16], focused only on specific diagnoses [17], or had short-term follow-up time for high-risk groups [18]. Therefore, larger population-based studies with longer follow-up time are needed to further evaluate the relationship between CHDs and poor neurodevelopmental outcomes. The purpose of this study was to determine the risk of ID, ID severity, and autism among children with CHDs in a population-based study.

## Methods

We conducted a retrospective cohort study based on singleton live births in Western Australia between January 1, 1983 and December 31, 2010. Records of all cases of CHD in liveborn infants were ascertained from the Western Australian Register of Developmental Anomalies (WARDA). The WARDA is a mandatory, active, statewide, population-based birth defects surveillance system that ascertains cases of birth defects in state residents from multiple sources including hospitals and clinics, inpatients and outpatients, private and public services. All major and minor birth defects diagnosed before pregnancy or after birth and up to 6 years of age are eligible for inclusion in the Register. All defects are classified using the British Paediatric Association (BPA) 5-digit extension of the International Classification of Disease (ICD), Ninth Revision System [19]. CHDs are classified using the BPA codes in the range of 745.00 to 747.90. A maximum number of 10 defects is recorded for each infant and syndromes are coded in conjunction with the major individual defects occurring in the infant. Records of liveborn children without a CHD notification to WARDA were randomly selected from the Western Australian birth register and were 2:1 frequency-matched by year of birth to the CHD cases to be used as a comparison cohort.

Western Australia established a Midwives Notification System (MNS) in 1975 to document information about all births attended by midwives. Data in both cohorts (the WARDA and MNS) were linked to MNS data to obtain information on maternal and infant characteristics and demographics. The data were also linked to the Intellectual Disability Exploring Answers (IDEA) database [20], a population-based registry that ascertains cases of intellectual disability through the Western Australian Disability Services Commission and the Western Australian Department of Education. Children with evidence of an ID before 18 years of age are eligible for inclusion in IDEA [21]. Eligibility criteria for cases from the Disability Services Commission included general intelligence to be more than 2 standard deviations below the mean for their age (or their full-scale intelligence quotient [IQ] was < 70) and deficiency in adaptive behaviors occurring before the age of 18 years [22]; or having a known condition consistent with an ID (such as Down syndrome). Children too young to be tested on a formal measure of intelligence were eligible for inclusion if a developmental test such as the Griffiths assessment scored them < 70, and they were considered vulnerable to ID. The linkage of subject-specific information was conducted by the Data Linkage Unit of the Western Australian Department of Health [23].

CHD was classified in two ways. The first was as a dichotomized variable (‘No CHD’ vs ‘CHD’) and the second was based on CHD severity: ‘No CHD,’ ‘Mild CHD’, ‘Moderate CHD’ or ‘Severe CHD’ (categories based on a modified classification by Marino et al. [7] and Warnes et al. [24]). Based on their BPA codes, CHDs were classified as mild, moderate, and severe. Detailed classification and corresponding ICD codes can be found in the Appendix. For those children with multiple CHD diagnoses, the most severe CHD diagnosis was used to determine their category. The ‘No CHD’ group was the reference for the multivariable regressions.

Individuals with ID from the Disability Services Commission were categorized into mild (IQ 55–69), moderate (IQ 40–54), or severe (IQ < 40). However, since the Department of Education did not differentiate between mild and moderate forms of ID; all records were categorized into one of three categories: mild/moderate, severe, or unknown ID. Those with unknown severity of ID (*n* = 36) were subsequently grouped with mild/moderate ID given that severe cases would have likely already been coded as severe.

The diagnostic categories for ID were defined as (1) autism with co-occurring ID, (2) known biomedical diagnosis ((e.g. Fragile X, maternal alcohol, or postnatal injury)) [25] without Down syndrome, (3) unknown cause with mild or moderate ID, and (4) no ID. Cases with a condition related to ID, but without a definite cause (e.g., environmental deficiency or prematurity) and those with no medical information available were classified as an unknown cause. Infants with Down syndrome (*n* = 303) were excluded from the primary analysis since Down syndrome is a major cause of ID and children with Down syndrome are more likely to have a CHD [26].

Maternal and infant covariates were obtained from the MNS. Infant characteristics included sex (male, female), grouped birth years (1983–1989, 1990–1999, 2000–2010), and gestational age (very preterm [20–31 completed weeks], moderate or late preterm [32–36 completed weeks], early term or term [≥37 completed weeks]). Maternal characteristics included: age at birth of the index child (< 20, 20–34, ≥35 years), race (Aboriginal, Caucasian, Other), and marital status (Married, Not Married [never married, separated, divorced, or widowed]).

The CHD (binary and severity categories) and infant/maternal covariates were analyzed against outcomes that corresponded to ID. Binary logistic regressions were performed for each of ID outcome with inclusion of only CHD (either binary or severity categories) in the model with and without adjustment for infant/maternal characteristics. We used odds ratios (OR) to approximate risk ratios since the probability of ID was very low in our study population (3.2%). Separate regressions were implemented for binary CHD (yes or no) and severity of CHD (None, Mild, Moderate or Severe) for each ID outcome. Potential confounders including maternal race [27, 28], infant sex [29, 30], infant gestational age [31, 32], maternal age [33, 34], and marital status [35] were identified from the scientific literature based on their association with both CHD and ID. Birth period was included in the model to adjust for possible time trend effects. Given small samples among children with any ID, each logistic regression model implemented Firth's penalization [36], minimizing the small-sample bias of maximum likelihood coefficients. Since Down syndrome is a known cause of ID, we conducted sensitivity analyses by including children with Down syndrome in separate analyses. Data analyses were conducted using SAS v9.4 (SAS Institute; Cary, NC).

The study was reviewed and approved by the Institutional Review Board at the University of Arkansas for Medical Sciences and by the Government of Western Australia Department of Health, Human Ethics Review Committee. Approval from the Western Australian Aboriginal Health Ethics Committee was also obtained. Consent was waived by the University of Arkansas for Medical Sciences the Institutional Review Board.

## Results

There were 792,373 births in Western Australia between January 1, 1983 and December 31, 2010. After random sampling and frequency matching, our population contained 20,592, of which 6563 (32%) had a CHD. Table 1 displays CHD status and classifications for children by ID status and different ID category and diagnostic information. Among children with any CHD, 7.1% had any type of ID; 6.3% had mild or moderate ID, while less than 1% had a severe ID. A greater percentage of children with CHDs had unknown cause of ID (3.5%), followed by biomedical (3.2%) and autism with co-occurring ID (0.4%).

**Table 1:** Congenital heart defect (CHD) status and classification for children with and without intellectual disabilities (IDs) in Western Australia, Australia, 1983–2010

	Intellectual Disability (ID)		Levels of Intellectual Disability			Diagnostic Categories			
	No ID^**a**^ *(n = 19,939)*	Any ID^**a**^ *(n = 653)*	None^**a**^ *(n = 19,939)*	Mild/Moderate^**a**^ *(n = 595)*	Severe^**a**^ *(n = 58)*	None^**a**^ *(n = 19,939)*	Autism with co-occurring ID *(n = 60)*	Biomedical^**a**^ *(n = 215)*	Unknown^**a**^ *(n = 378)*
	n (%)	n (%)	n (%)	n (%)	n (%)	n (%)	n (%)	n (%)	n (%)
**Congenital Heart Defects (CHD)**									
Yes	6097 (92.9)	466 (7.1)	6097 (92.9)	412 (6.3)	54 (0.8)	6097 (92.9)	26 (0.4)	208 (3.2)	232 (3.5)
No	13,842 (98.7)	187 (1.3)	13,842 (98.7)	183 (1.3)	4 (0.1)	13,842 (98.7)	34 (0.2)	7 (0.1)	146 (1.0)
**Classifications of CHD**									
None	13,915 (98.6)	197 (1.4)	13,915 (98.6)	189 (1.3)	8 (0.1)	13,915 (98.6)	35 (0.2)	13 (0.1)	149 (1.1)
Mild	444 (92.1)	38 (7.9)	444 (92.1)	30 (6.2)	8 (1.7)	444 (92.1)	3 (0.6)	17 (3.5)	18 (3.7)
Moderate	4911 (92.9)	373 (7.1)	4911 (92.9)	336 (6.4)	37 (0.7)	4911 (92.9)	19 (0.4)	161 (3.1)	193 (3.7)
Severe	669 (93.7)	45 (6.3)	669 (93.7)	40 (5.6)	5 (0.7)	669 (93.7)	3 (0.4)	24 (3.4)	18 (2.5)

^a^Sample size with binary CHD is 20,592. Because total of 83 children with certain isolated BPA codes were excluded from different CHD classifications but not from binary CHD, numbers in parenthesis present sample size for binary CHD. There were 14,112 children with no CHD, 482 children with mild CHD, 5284 children with moderate CHD and 714 children with severe CHD. Sample size for different CHD classification as follows, 653 children in ID, 595 children in mild/moderate ID, 58 children in severe ID, 60 children in autism with co-occurring ID, 159 children in biomedical ID, and 378 children in unknown ID. The totals do not add up due to the classifications

Almost 8% of children with mild CHD had an ID, 7.1% of children with a moderate CHD had an ID and 6.3% of children with severe CHD had an ID. Among children with moderate CHDs, 6.4% had a mild or moderate ID compared to mild CHDs (6.2%) and severe CHDs (5.6%) within the same ID category. Severe ID had a higher frequency among children with mild CHDs (1.7%). Among children with mild and moderate CHDs, the majority had an unknown cause of ID (3.7%), followed by a biomedical cause of ID (3.5 and 3.1%, respectively). A greater percentage of severe CHD had biomedical cause of ID (3.4%) followed by unknown cause of ID (2.5%).

Table 2 presents unadjusted and adjusted odds ratios (aORs) and 95% confidence intervals (CIs) for the risk of IDs. Compared to children without CHDs, children with any CHD had 5.26 times (95% CI 4.42, 6.26) the odds of having an ID and 4.76 times (95% CI 3.98, 5.70) the odds of having mild/moderate ID adjusting for maternal/infant characteristics. Children with any CHD had 24.66 times (95% CI 9.72, 62.57) the odds of having a severe ID; however, the model was very unstable. The odds of any ID was the greatest in children with mild CHDs (aOR 5.72, 95% CI 3.97, 8.25), followed by moderate and severe CHDs (aOR 5.28, 95% CI 4.40, 6.32 and aOR 4.47, 95% CI 3.20, 6.25, respectively). However, children with moderate CHDs had the highest odds (aOR 4.87, 95% CI 4.05, 5.86) of having mild/moderate ID. Children with mild CHDs had the highest odds (aOR 47.41, 95% CI 15.48, 145.22) of having a severe ID, followed by severe CHDs (aOR 22.08, 95% CI 6.63, 73.60). However, the sample size of children with a severe ID was very small and the model was very unstable.

**Table 2:** Odds ratios (OR) and 95% confidence intervals (CI) from unadjusted and adjusted logistic regression analyses for risk of intellectual disability (ID) severity, and diagnostic categories among children with and without congenital heart defects (CHDs), Western Australia, Australia, 1983–2010

Severity of ID		**Intellectual Disability (ID)**^**a**^ *(n = 653)*		**Mild/ Moderate ID**^**a**^ *(n = 595)*		**Severe ID**^**a**^ *(n = 58)*	
		**Unadjusted** **OR (95% CI)**	**Adjusted** **OR (95% CI)**	**Unadjusted** **OR (95% CI)**	**Adjusted** **OR (95% CI)**	**Unadjusted** **OR (95% CI)**	**Adjusted** **OR (95% CI)**
	**Congenital Heart Defects (CHD)**^**b**^						
	No CHD	Referent	Referent	Referent	Referent	Referent	Referent
	Any CHD	5.66 (4.76, 6.72)	5.26 (4.42, 6.26)	5.11 (4.28, 6.10)	4.76 (3.98, 5.70)	30.65 (11.10, 84.65)	24.66 (9.72, 62.57)
	**CHD Severity**^**b**^						
	No CHD	Referent	Referent	Referent	Referent	Referent	Referent
	Mild CHD	6.34 (4.41, 9.09)	5.72 (3.97, 8.25)	5.11 (3.44, 7.61)	4.66 (3.12, 6.96)	62.35 (18.71, 207.83)	47.41 (15.48, 145.22)
	Moderate CHD	5.62 (4.70, 6.72)	5.28 (4.40, 6.32)	5.18 (4.31, 6.21)	4.87 (4.05, 5.86)	26.07 (9.29, 73.18)	21.35 (8.30, 54.89)
	Severe CHD	4.98 (3.56, 7.00)	4.47 (3.20, 6.25)	4.52 (3.18, 6.42)	4.07 (2.86, 5.78)	25.86 (6.93, 96.53)	22.08 (6.63, 73.58)
Diagnostic Categories		**Autism with co-occurring ID**^**a**^ *(n = 60)*		**Biomedical ID**^**a**^ *(n = 215)*		**Unknown ID**^**a**^ *(n = 378)*	
		**Unadjusted** **OR (95% CI)**	**Adjusted** **OR (95% CI)**	**Unadjusted** **OR (95% CI)**	**Adjusted** **OR (95% CI)**	**Unadjusted** **OR (95% CI)**	**Adjusted** **OR (95% CI)**
	**CHD**^**b**^						
	No CHD	Referent	Referent	Referent	Referent	Referent	Referent
	Any CHD	1.74 (1.04, 2.90)	1.76 (1.07, 2.88)	67.46 (31.75, 143.35)	59.69 (28.99, 122.92)	3.61 (2.93, 4.45)	3.27 (2.65, 4.05)
	**CHD Severity**^**b**^						
	No CHD	Referent	Referent	Referent	Referent	Referent	Referent
	Mild CHD	2.75 (0.84, 8.99)	3.23 (1.11, 9.38)	75.71 (31.24, 183.49)	65.41 (27.76, 154.14)	3.84 (2.33, 6.33)	3.45 (2.09, 5.70)
	Moderate CHD	1.58 (0.90, 2.76)	1.61 (0.94, 2.76)	64.83 (30.40, 138.26)	57.81 (27.98, 119.47)	3.73 (3.00, 4.63)	3.43 (2.75, 4.27)
	Severe CHD	1.83 (0.56, 5.96)	2.14 (0.74, 6.18)	70.94 (30.46, 165.22)	60.81 (26.92, 137.37)	2.55 (1.55, 4.19)	2.25 (1.38, 3.68)

^a^Because 83 children with certain isolated BPA codes were excluded from different CHD classifications but not from binary CHD, numbers in parenthesis display sample sizes for binary CHD. Sample sizes for different CHD classification are as follows, 643 children in ID, 589 children in mild/moderate ID, 54 children in severe ID, 59 children in autism with co-occurring ID, 209 children in biomedical ID, and 375 children in unknown ID

^b^Separate models were computed first treating CHD as a binary independent variable and then using different classifications of CHD. Both models adjusted for maternal race, infant sex, infant gestational age, maternal age, marital status, and birth time period

Children with any CHD had 1.76 times the odds of having autism (95% CI 1.07, 2.88), and 3.27 times the odds of having an unknown cause of ID (95% CI 2.65, 4.05) compared to children without CHD, after adjustment for maternal/infant covariates using Firth’s method [36]. The odds of having autism was the greatest for children with mild CHD (aOR 3.23, 95% CI 1.11, 9.38). Similarly, children with mild CHD had the highest odds of having an unknown cause of ID (aOR 3.45, 95% CI 2.09, 5.70).

When we included children with Down syndrome in our analyses (*n* = 303), the same overall pattern was observed except among children with mild and moderate CHDs: 13.2% of children with mild CHDs had mild or moderate ID compared to children with moderate CHDs (10.1%) (Supplemental Table 1). We observed similar associations when including children with Down syndrome into the analytical sample except for children with mild CHDs, which had the greatest odds of having mild/moderate ID (aOR 10.92, 95% CI 8.13, 14.68) A similar pattern was observed when children with Down syndrome included in the analytical sample except that children with mild CHDs had the greatest odds (aOR 10.92, 95% CI 8.13, 14.68) of having mild/moderate ID (Supplemental Table 2). In our sensitivity analysis, all children with Down syndrome had some form of CHD; therefore, all adjusted estimates remained the same (Supplemental Table 2).

## Discussion

The purpose of this study was to determine the risk of ID, ID severity, and autism among children with CHDs overall and by CHD severity. Children with any CHD had about a five-fold increased odds of developing an ID; three-fold greater odds of ID with unknown cause; and nearly two-fold increased odds of autism compared to children without CHDs. We observed that the odds of ID tend to be slightly lower for severe CHD in all categories of ID, but the confidence intervals are wide.

Greater odds of ID applied to all severities of CHDs. Among pediatric patients with complex CHDs, there is a distinctive pattern of neurodevelopmental and behavioral deficits characterized by impaired mild cognition, social interaction, core communication skills, including pragmatic language, as well as inattention, impulsive behavior, and impaired executive function [37]. Children with severe CHDs may have significantly lower intellectual functioning than children with mild or moderate CHDs [38].

Despite great advances in screening and diagnosis [39], in rare cases, CHDs may go undetected into adolescence or early adulthood resulting in misclassification of children with CHDs. However, using data from a statewide, population-based, active birth defect surveillance system that ascertains nearly all cases of birth defects from multiple sources, we feel that the number would be small. The identification of ID in the population is through ascertainment from both Disability Services and educational support services, therefore minimizing the possibility that children with a CHD will be more likely to obtain an ID diagnosis through their medical care. Children with CHDs that undergo medical and surgical therapies have been shown to be at a higher risk for morbidities, including physical, psychological, and neurodevelopmental functioning [40]. However, there have been conflicting studies conducted examining the association between surgical and neurodevelopmental outcomes later in life [41, 42]. A recent study found that children with CHDs receive special education services more often than children without birth defects [43], which may explain differences observed among different levels of CHD severity. In a study evaluating early childhood survivors of CHD, all children with serious CHDs were found to be at high risk of motor problems regardless of surgical procedures or cyanotic status [44]. It is important to consider the implications of these medical and surgical therapies in this specific pediatric population to ensure that these children have access to age-appropriate neurodevelopmental assessments and, where necessary, interventions and therapies throughout childhood and adulthood. The patterns of neurodevelopment during the life course requires a deeper knowledge, and is crucial, since certain interventions and individuals (e.g., parents, schools, medical providers, etc.) may have a greater influences at specific time periods [45].

A major strength of this study is the large study population and use of data from WARDA, which utilizes active surveillance methods, allowing for almost complete ascertainment of cases with CHDs. Additionally, the IDEA database provides a near-complete ascertainment and study population of children with IDs, compared to using clinic- or hospital-based registries. One potential limitation is the lack of information on operative and surgical variables that may explain the differences observed in the severities of CHDs. Additionally, our information on intellectual disability diagnosis may be limited and our classification of unknown cause may not reflect a later genetic diagnosis. Also, our study did not include data such as functioning level of the child, amount and type of support services received by families, parental education, and age of diagnosis.

Our results contribute to the current literature that children with CHDs have increased odds of ID and autism co-occurring with ID. Further development of early interventions to optimize neurodevelopmental outcomes in children with CHDs in Western Australia is critically needed to optimize long-term outcomes for these children and their families. Further research is needed to examine the underlying etiology of neurodevelopmental disabilities and outcomes in children with CHDs.

## Conclusions

There is a need for effective clinical surveillance for children with CHDs, including raising awareness in physicians, other healthcare professionals, and organizations. Children with CHDs constitute an at-risk population that requires follow-up visits to improve early detection of neurodevelopmental outcomes and to reduce disease burden.

## Supplementary Information

**Additional file 1.**

**Additional file 2.**

## Data Availability

The datasets generated during and analyzed during the current study are not publicly available due to requirements of the IDEA data registry and Government of Western Australia but data are available with appropriate human subject approvals from the corresponding author on reasonable request.

## Abbreviations

CHDs: Congenital heart defects
ID: Intellectual disability
WARDA: Western Australian Register of Developmental Anomalies
BPA: British Paediatric Association
ICD: International Classification of Disease
MNS: Midwives Notification System
IDEA: Intellectual Disability Exploring Answers
OR: Odds ratio
aOR: Adjusted odds ratio
CI: Confidence interval
