# Supplementary material for: Intellectual disabilities and autism among children with congenital heart defects, Western Australia, 1983–2010
Source: BMC Pediatr. 2023 Mar 4;23:106. doi: 10.1186/s12887-023-03924-3 (PMC9985207; doi:10.1186/s12887-023-03924-3)
Supplement: Supplementary file 1 — Additional file 1. [file 12887_2023_3924_MOESM1_ESM.docx]

**Appendix**

| **British Pediatric Association (BPA) Code** | **Congenital Heart Defect** |
| --- | --- |
| **Mild CHD** |  |
| 745.50 | Ostium secundum type atrial septal defect |
| 745.58 | Other specified atrial septal defect |
| 745.59 | Atrial septal defect, not otherwise specified |
| 746.47 | Bicuspid aortic valve |
| 746.49 | Unspecified anomalies of the aortic valves |
| 747.25 | Vascular ring (aorta) |
| **Moderate CHD** |  |
| 745.20 | Tetralogy of Fallot |
| 745.21 | Tetralogy of Fallot plus atrial septal defect (also labeled as pentalogy of Fallot) |
| 745.40 | Ventricular septal defect (VSD) |
| 745.48 | Perimembranous VSD/Muscular VSD/Inlet VSD |
| 745.49 | VSD, not otherwise specified |
| 745.60 | Ostium primum defect |
| 745.61 | Single common atrium |
| 745.62 | Common atrioventricular canal with VSD |
| 745.63 | Common atrioventricular canal |
| 745.68 | Other specified cushion defect |
| 745.69 | Endocardial cushion defect, unspecified type |
| 746.00 | Atresia of pulmonary valve, congenital |
| 746.01 | Stenosis of pulmonary valve |
| 746.02 | Stenosis of pulmonary valve, congenital |
| 746.08 | Other specified anomalies of pulmonary valve |
| 746.09 | Unspecified anomaly of pulmonary valve |
| 746.20 | Ebstein's anomaly |
| 746.30 | Congenital stenosis of aortic valve |
| 746.31 | Aortic valve dysplasia |
| 746.40 | Aortic valve insufficiency or regurgitation, congenital |
| 746.51 | Mitral valve cleft |
| 746.52 | Mitral valve leaflet (single) |
| 746.53 | Mitral valve anomaly |
| 746.55 | Mitral valve anomaly (NOS) |
| 746.56 | Mitral valve leaflet anomaly |
| 746.84 | Trilogy of Fallot |
| 746.87 | Congenital heart block |
| 746.88 | Anomalies of coronary artery or sinus |
| 747.00 | Patent ductus arteriosus |
| 747.01 | Duct arteriosus prem closure |
| 747.10 | Preductal (proximal) coarctation of aorta |
| 747.11 | Postductal (distal) coarctation of aorta |
| 747.12 | Aortic arch (interrupted) |
| 747.18 | Coarctation of aorta (other) |
| 747.19 | Unspecified coarctation of aorta |
| 747.21 | Hypoplasia of aorta |
| 747.22 | Supra-aortic stenosis (supravalvular) |
| 747.24 | Aneurysm of sinus of Valsalva |
| 747.28 | Interrupted aortic arch, not otherwise specified, type not specified |
| 747.32 | Pulmonary artery stenosis/Peripheral pulmonary artery stenosis |
| 747.42 | Total anomalous pulmonary venous return (TAPVR) |
| 747.43 | Partial anomalous pulmonary venous return |
| 747.48 | Other specified anomalies of great veins |
| 747.49 | Unspecified anomalies of great veins |
| **Severe CHD** |  |
| 745.00 | Common truncus |
| 745.01 | Aortic Septal Defects |
| 745.10 | Transposition of great vessels, complete (with no VSD) |
| 745.11 | Transposition of great vessels, incomplete (with VSD) |
| 745.12 | Corrected transposition of great vessels |
| 745.13 | Double outlet right ventricle (DORV) with normally related great vessels |
| 745.14 | DORV with transposed great vessels |
| 745.15 | DORV, relationship of great vessels not otherwise specified |
| 745.18 | Other specified transposition of great vessels, no mention of DORV |
| 745.19 | Unspecified transposition of great vessels |
| 745.30 | Common ventricle |
| 745.41 | Eisenmenger’s Syndrome |
| 745.80 | Other bulbus cordis anomalies and anomalies of cardiac septal closure |
| 746.10 | Tricuspid valve atresia |
| 746.10 | Tricuspid valve insufficiency or regurgitation, congenital/Tricuspid stenosis or hypoplasia |
| 746.12 | Tricuspid Valve Regurgitation |
| 746.18 | Other anomalies of the tricuspid valve |
| 746.70 | Hypoplastic left heart syndrome |
| 746.71 | Hypoplastic right heart syndrome |
| 746.72 | Hypoplastic left ventricle syndrome |
| 746.77 | Atrium and/or ventricle dilated |
| 746.82 | Cor Triatriatum |
| 746.83 | Pulmonary infundibular (subvalvular) stenosis |
| 746.85 | Anomalies of pericardium |
| 746.88 | Heart anomaly other specified: Hypoplastic left ventricle/Includes: Hypoplastic right heart (ventricle), Hypoplastic ventricle, not otherwise specified, Ventricular hypertrophy, unilateral (left or right) |
| 746.99 | "Pulmonic" or "pulmonary" atresia, stenosis, or hypoplasia, not otherwise specified (no mention of valve or artery) |
| 747.20 | Atresia of aorta |
| 747.21 | Interrupted aortic arch, Type A/Interrupted aortic arch, Type B/Interrupted aortic arch, Type C |
| 747.30 | Pulmonary artery atresia, absence, or agenesis |
| 747.31 | Pulmonary artery atresia with septal defect |
| **Exclude from Classification but not the study** |  |
| 746.50 | Congenital mitral valve stenosis |
| 746.60 | Mitral valve insufficiency or regurgitation, congenital |
| 746.80 | Other specified anomalies of the aortic valves |
| 746.80 | Dextrocardia without situs inversus |
| 746.86 | Anomalies of myocardium |
| 746.88 | Other specified anomalies of heart/Other defects of the atria |
| 746.90 | Unspecified anomalies of heart valves |
| 746.91 | Anomalous bands of heart |
| 746.92 | Acyanotic congenital heart disease, not otherwise specified |
| 746.93 | Cyanotic congenital heart disease, not otherwise specified |
| 746.99 | Unspecified anomaly of heart |
| 747.26 | Overriding aorta |
| 747.27 | Congenital aneurysm of aorta |
| 747.28 | Other specified anomalies of aorta |
| 747.29 | Unspecified anomalies of aorta |
| 747.33 | Aneurysm of pulmonary artery |
| 747.34 | Pulmonary arteriovenous malformation or aneurysm |
| 747.38 | Other specified anomaly of pulmonary artery |
| 747.39 | Unspecified anomaly of pulmonary artery |
| 747.40 | Stenosis of vena cava (inferior or superior) |
| 747.80 | Other specified anomalies of circulatory system |
| 747.81 | Other specified anomalies of circulatory system |
| **Exclude from study** |  |
| 747.41 | Persistent left superior vena cava |
| 747.44 | Anomalous portal vein termination |
| 747.45 | Portal vein - hepatic artery fistula |
| 747.23 | Persistent right aortic arch |
| 747.61 | Other anomalies of renal artery |
| 747.62 | Arteriovenous malformation (peripheral) |
| 747.64 | Other anomalies of peripheral arteries |
| 747.65 | Other anomalies of peripheral veins |
| 747.68 | Other anomalies of peripheral vascular system |
