# Supplementary material for: Intellectual disabilities and autism among children with congenital heart defects, Western Australia, 1983–2010
Source: BMC Pediatr. 2023 Mar 4;23:106. doi: 10.1186/s12887-023-03924-3 (PMC9985207; doi:10.1186/s12887-023-03924-3)
Supplement: Supplementary file 2 — Additional file 2. [file 12887_2023_3924_MOESM2_ESM.docx]

**Supplemental Table 1.** Sensitivity analysis for Congenital heart defect (CHD) status and classification for children with and without intellectual disabilities (IDs) including children with Down syndrome in Western Australia, 1983-2010

|  | **ID** | | **Severity of Intellectual Disability** | | | **Diagnostic Categories** | | | | |
| --- | --- | --- | --- | --- | --- | --- | --- | --- | --- | --- |
|  | **No ID^a^**  *(n=19,939)* | **Any ID^a^**  *(n=956)* | **None^a^**  *(n=19,939)* | **Mild/Moderate^a^**  *(n=872)* | **Severe^a^**  *(n=84)* | **None^a^**  *(n=19,939)* | **Autism with co-occurring ID^a^**  *(n=60)* | **Biomedical^a^**  *(n=215)* | **Down Syndrome^a^**  *(n=303)* | **Unknown^a^**  *(n=378)* |
|  | **n (%)** | **n (%)** | **n (%)** | **n (%)** | **n (%)** | **n (%)** | **n (%)** | **n (%)** | **n (%)** | **n (%)** |
| **CHD**  Yes  No | 6097 (88.8)  13842 (98.7) | 769 (11.2)  187 (1.3) | 6097 (88.8)  13842 (98.7) | 689 (10)  183 (1.3) | 80 (1.2)  4 (<0.1) | 6097 (88.8)  13842 (98.7) | 26 (0.4)  34 (0.2) | 208 (3.2)  7 (0.1) | 303 (4.4)  0 (0.0) | 232 (3.4)  146 (1) |
| **Classifications of CHD**  None  Mild  Moderate  Severe | 13915 (98.6)  444 (83.9)  4911 (88.9)  669 (91.9) | 198 (1.4)  85 (16.1)  614 (11.1)  59 (8.1) | 13915 (98.6)  444 (83.9)  4911 (88.9)  669 (91.9) | 190 (1.3)  70 (13.2)  558 (10.1)  54 (7.4) | 8 (0.1)  15 (2.8)  56 (1)  5 (0.7) | 13915 (98.6)  444 (83.9)  4911 (89.9)  669 (91.9) | 35 (0.2)  3 (0.6)  19 (0.3)  3 (0.4) | 13 (0.1)  17 (3.5)  161 (3.1)  24 (3.4) | 1 (0.0)  47 (8.9)  241 (4.4)  14 (1.9) | 149 (1.1)  18 (3.4)  193 (3.5)  18 (2.5) |

^a^ *After including children with Down syndrome, sample size with binary CHD is 20,895. Because total of 83 children with certain isolated BPA codes were excluded from different CHD classifications but not from binary CHD, numbers in parenthesis present sample size for binary CHD. There were 14,029 children with no CHD, 529 children with mild CHD, 5,525 children with moderate CHD and 728 children with severe CHD. Sample size for different CHD classification as follows, 956 children in ID, 872 children in mild/moderate ID, 84 children in severe ID, 60 children in autism with co-occurring ID, 215 children in biomedical ID, 303 children in Down syndrome, and 378 children in unknown ID. The totals do not add up due to the classifications*

**Supplemental Table 2.** Logistic regression analyses for intellectual disability (ID), ID severity, and diagnostic categories among children with and without congenital heart defects (CHDs) including Down syndrome.

| Severity of ID |  | ID^a^  *(n=956)* | | Mild/ Moderate ID^a^  *(n=872)* | | Severe ID^a^  *(n=84)* | |
| --- | --- | --- | --- | --- | --- | --- | --- |
|  |  | **Unadjusted**  **OR (95% CI)** | **Adjusted**  **OR (95% CI)** | **Unadjusted**  **OR (95% CI)** | **Adjusted**  **OR (95% CI)** | **Unadjusted**  **OR (95% CI)** | **Adjusted**  **OR (95% CI)** |
|  | **CHD^b^**  No CHD  Any CHD | Referent  5.65 (4.76, 6.71) | Referent  8.73 (7.42, 10.29) | Referent  5.10 (4.28, 6.09) | Referent  8.03 (6.79, 9.48) | Referent  27.49 (10.51, 71.94) | Referent  36.56 (14.53, 92.48) |
|  | **CHD Severity^b^**  No CHD  Mild CHD  Moderate CHD  Severe CHD | Referent  14.20 (10.81, 18.66)  9.24 (7.82, 10.91)  6.56 (4.85, 8.87) | Referent  12.91 (9.78, 17.05)  8.77 (7.42, 10.37)  5.87 (4.33, 7.96) | Referent  11.97 (8.95, 16.00)  8.58 (7.24, 10.17)  6.14 (4.49, 8.39) | Referent  10.92 (8.13, 14.68)  8.17 (6.89, 9.70)  5.52 (4.04, 7.56) | Referent  107.240 (37.38, 307.67)  35.38 (13.54, 92.44)  25.26 (7.25, 88.06) | Referent  90.94 (32.23, 256.66)  32.68 (12.80, 83.40)  21.28 (6.29, 71.95) |
| Diagnostic Categories |  | **Autism with co-occurring ID)^a^**  *(n=60)* | | **Biomedical ID^a^**  *(n=215)* | | **Unknown ID^a^**  *(n=378)* | |
|  |  | **Unadjusted**  **OR (95% CI)** | **Adjusted**  **OR (95% CI)** | **Unadjusted**  **OR (95% CI)** | **Adjusted**  **OR (95% CI)** | **Unadjusted**  **OR (95% CI)** | **Adjusted**  **OR (95% CI)** |
|  | **CHD^b,c^**  No CHD  Any CHD | Referent  1.74 (1.05,2.90) | Referent  1.76 (1.07, 2.88) | Referent  63.11 (30.44,130.84) | Referent  59.69 (28.99, 122.92) | Referent  3.60, (2.92, 4.44) | Referent  3.27 (2.65, 4.05) |
|  | **CHD Severity^b,c^**  No CHD  Mild CHD  Moderate CHD  Severe CHD | Referent  3.16 (1.05, 9.54)  1.59 (0.91, 2.78)  2.10 (0.70, 6.32) | Referent  3.23 (1.11, 9.38)  1.61 (0.94, 2.76)  2.14 (0.74, 6.18) | Referent  72.66 (30.72, 171.87)  60.69 (29.16, 126.29)  67.54 (29.70, 153.62) | Referent  65.41 (27.76, 154.14)  57.81 (27.98, 119.47)  60.81 (26.92, 137.37) | Referent  3.93 (2.40, 6.44)  3.72 (3.00, 4.63)  2.61 (1.60, 4.26) | Referent  3.45 (2.09, 5.70)  3.43 (2.75, 4.27)  2.25 (1.38, 3.68) |

*^a^ Because total of 83 children with certain isolated BPA codes were excluded from different CHD classifications but not from binary CHD, numbers in parenthesis present sample size for binary CHD. Sample size for different CHD classification as follows, 643 children in ID, 589 children in mild/moderate ID, 58 children in severe ID, 59 children in known genetic condition and unknown etiology ID (i.e., autism with co-occurring ID), 209 children in biomedical ID, and 375 children in unknown ID.*

*^b^ Separate models were ran first treating CHD as a binary independent variable and then as different classifications of CHD. Both models adjusted for maternal race, infant sex, infant gestational age, maternal age, marital status, and birth timeframe.*

*^c^ Because all children with the outcome of Down syndrome had also CHDs, this outcome was not modeled.*
